# Long-lasting disease stabilization in the absence of toxicity in metastatic lung cancer patients vaccinated with an HLA-A2-restricted epitope derived from indoleamine 2,3 dioxygenase

**DOI:** 10.1186/2051-1426-1-S1-P273

**Published:** 2013-11-07

**Authors:** Trine Z Iversen, Lotte Engell-Noerregaard, Eva Ellebaek, Rikke Andersen, Stine K Larsen, Jon Bjoern, Claus Zeyher, Cécile Gouttefangeas, Birte M Thomsen, Bente Holm, Per thor Straten, Anders Mellemgaard, Mads H Andersen, Inge M Svane

**Affiliations:** 1 Center for Cancer Immune Therapy, Department of Haematology, Copenhagen University Hospital at Herlev, Herlev, Denmark; 2 Department of Oncology, Copenhagen University Hospital at Herlev, Herlev, Denmark; 3 Interfaculty Institute of Biochemistry, University of Tübingen, Tübingen, Germany; 4 Interfaculty Institute of Cell Biology Department of Immunology, University of Tübingen, Tübingen, Germany; 5 Department of Pathology, Bispebjerg Hospital, Copenhagen, Denmark

## Purpose

To investigate targeting of indoleamine 2.3 dioxygenase (IDO) enzyme using a synthetic peptide vaccine administered to patients with metastatic non small-cell lung cancer (NSCLC).

## Experimental design

In a clinical phase I study we treated 15 HLA-A2 positive patients with stage III-IV NSCLC in disease stabilization (SD) after standard chemotherapy. Patients were treated with Imiquimod ointment and subcutaneous vaccinations (100 µg IDO5 peptide, sequence ALLEIASCL, formulated in 900 µL Montanide). Primary end point was toxicity. Clinical benefit and immunity were assessed as secondary endpoints.

## Results

No severe toxicity was observed. One patient developed a partial response (PR) after 1 year of vaccine treatment while long-lasting disease stabilization (SD≥8.5 months) was demonstrated in another 6 patients. The median overall survival (OS) was 25.9 months. Patients demonstrated significant improved OS (P=0.03) when compared to the group of patients excluded due to HLA-A2 negativity. IDO specific CD8+ T cell immunity was demonstrated by IFN-γ Elispot and Tetramer staining. FACS analyses demonstrated a significant reduction of the Treg population (P=0.03) and a significant increase in NK cell population (P=0.05) after the 6th vaccine (2.5 months) compared to pre-treatment levels. Furthermore, expression of IDO was detected in 9/10 tumour biopsies by immunohistochemistry. HPLC analyses of Kynurenine/Tryptophan (Kyn/Trp) ratio in sera were performed. In long term analyses of two clinical responding patients the ratio of Kyn/Trp remained stable.

## Conclusion

The vaccine was well-tolerated with no severe toxicity occurring. A median OS of 25.9 months was demonstrated and long-lasting PR+SD were seen in 47% of the patients.

